# Extracellular Nucleosomes Accelerate Microglial Inflammation *via* C-Type Lectin Receptor 2D and Toll-Like Receptor 9 in mPFC of Mice With Chronic Stress

**DOI:** 10.3389/fimmu.2022.854202

**Published:** 2022-06-29

**Authors:** Huanghui Wu, Han Bao, Cong Liu, Qiao Zhang, Ailing Huang, Minxue Quan, Chunhui Li, Ying Xiong, Guozhong Chen, Lichao Hou

**Affiliations:** ^1^ Department of Anesthesiology, Xiang’an Hospital of Xiamen University, School of Medicine, Xiamen University, Xiamen, China; ^2^ Department of Anesthesiology and Perioperative Medicine, Shanghai Fourth People’s Hospital Affiliated to Tongji University, Shanghai, China

**Keywords:** extracellular nucleosomes, microglia, C-type lectin receptor 2D, toll-like receptor 9, inflammation, chronic stress

## Abstract

Damage-associated molecular patterns (DAMPs) are the primary promoter of progressive neuroinflammation and are associated with chronic stress-related emotional disorders. The present study investigated the role and mechanism of extracellular nucleosomes and histones, the newly defined DAMPs, in mice with chronic stress. C57BL/6 mice were exposed to chronic unpredictable mild stress (CUMS) and corticosterone drinking, respectively, for 4 weeks. Negative emotional behaviors were comprehensively investigated. Microglial morphology, oxidative stress, and inflammation, as well as C-type lectin receptor 2D (Clec2d) and Toll-like receptor 9 (TLR9) expression in medial prefrontal cortex (mPFC) were assessed with flow cytometer and cell sorting. Specifically, microglial pro-inflammatory activation and inflammation were further investigated with stereotactic injection of recombinant nucleosomes and histones in mPFC and further evaluated with AAV-Clec2d knocking-down, DNase I, and activated protein C (APC) pretreatment. Moreover, the rescue effect by AAV-Clec2d knocking-down was observed in mice with chronic stress. Mice with chronic stress were presented as obviously depressive- and anxiety-like behaviors and accompanied with significant microglial oxidative stress and inflammation, indicating by reactive oxygen species (ROS) production, primed nuclear factor-κB (NF-κB) signaling pathway, activated NACHT, LRR, and PYD domain–containing protein 3 (NLRP3) inflammasome, and upregulated Clec2d and TLR9 in mPFC, together with histones dictation in cerebrospinal fluid and extracellular trap formation. Stereotactic injection of nucleosomes was contributed to promote microglial inflammation rather than histones in mPFC, indicating that the pro-inflammatory role was derived from extracellular histones-bound DNA but not freely histones. AAV-Clec2d knocking-down, DNase I, and APC were all effective to inhibit nucleosome-induced microglial oxidative stress and inflammation. Moreover, AAV-Clec2d knocking-down in mice with chronic stress exhibited reduced microglial inflammation and improved negative emotional behaviors. Our findings reveal a novel mechanism of DAMP-associated inflammation that extracellular nucleosomes accelerate microglial inflammation *via* Clec2d and TLR9, and then contribute to chronic stress-induced emotional disorders.

## Introduction

There is a growing body of evidence confirming the bidirectional association between amplified neuroinflammation and chronic stress–related emotional disorders (1, 2). Microglia, a dynamic and active participant in the central nervous system (CNS), play a critical role in maintaining brain homeostasis and surveil the specialized niche of the brain parenchyma for disruptions in homeostasis (3). As an exclusive resident immune–competent cell in the CNS, microglia are capable of secreting an array of pro- and anti-inflammatory cytokines under physiological conditions, such as chronic stress (3, 4). Activated microglia extensively participate in removing cellular debris and apoptotic or necrotic cells by phagocytosis, lending trophic support to neurons and modulating neuronal activity, as well as modifying synaptic connections and plasticity (5). However, previous clinical studies revealed that the overactivated microglia with production of pro-inflammatory cytokines such as tumor necrosis factor–α, interleukin (IL)-1β, and IL-6, are closely associated with the occurrence and severity in patients with neuropsychiatric disorder (6–8). Therefore, microglia have been considered as the dominant mediator, which aggravates and propagates neuroinflammation, contributing to the development of chronic stress-related emotional disorders. An interesting line of evidence suggests that damage-associated molecular patterns (DAMPs) released from damaged and necrotic cell were contributed to the neuroinflammatory process *via* Toll-like receptors (TLRs) (9, 10). However, the origination of neuroinflammation during chronic stress is still remained uncertain (11).

Nucleosome, a repeating structural unit of chromatin that contains histones and DNA, is the basic unit for higher-order chromatin structure and acts as specialized transcription factory in the nucleus. All nucleosome components, including freely histones, DNA, and histones-bound DNA, will be released into the extracellular space during cellular stress and necrosis (12, 13). Extracellular freely histones and DNA are the major DAMPs that induce cytotoxicity and pro-inflammatory signaling through TLR2 and TLR4, as well as TLR9 and intracellular nucleic acid sensing mechanisms, respectively (14). More recently, C-type lectin receptor 2D (Clec2d) has been demonstrated as a dedicated receptor for histones by recognizing sulfated polysaccharides through their basic N-terminal region, providing a novel fascinating avenue for cellular stress and tissue damage sensing pathway (15, 16). However, the pro-inflammatory signaling induced by extracellular nucleosomes has not been studied as extensively as the separate effects brought about by freely histones and DNA, especially in microglia-derived inflammation during chronic stress. Therefore, we designed the present study to investigate the pro-inflammatory role and mechanism of extracellular nucleosomes and histones to microglia in mice with chronic stress. Additionally, the potential effect of microglia-derived inflammation on spine pruning was discussed.

## Methods and Materials

### Animals

Adult male C57BL/6 mice, aged 6~8 weeks, weighted 18~22 g, were housed in the core animal facility with ambient temperature of 22°C ± 1°C, 30~70% humidity, 12-h dark/12-h light cycle (light on at 07:00 a.m.), and food and water *ad libitum* in Xiamen University. Experimental protocol and all procedures were received prior approval from the Institutional Animal Care and Use Committee at Xiamen University (Xiamen, China, XMULAC20190054). Efforts were made to minimize animal suffering and to reduce the number of animals used.

### Chronic Stress Protocol

Mice were randomly assigned to receive chronic unpredictable mild stress (CUMS), corticosterone (CORT) drinking, or control group. CUMS protocol was involved a variety of mild stressors, including 24 h of food deprivation, 24 h of water deprivation, 1 h of exposure to empty bottles, 8 h of cage tilt (30°), overnight illumination, 24 h of habitation in a soiled cage (200 ml of water in 100 g of sawdust bedding), 5 min of forced swimming (FS) at 8°C, 2 h of physical restraint, and 24 h of exposure to a foreign object (e.g., a piece of plastic) (17). Mice in CUMS group were daily received two of those stressors combination that were prior randomly scheduled for a 4-week period. Mice in CORT group were received CORT drinking water daily for a 6-week period. CORT (Sigma-Aldrich, St. Louis, MO, USA) was dissolved in ethanol absolute (Sigma-Aldrich) and mixed with potable water at a final concentration of 0.1 mg/ml CORT and 1% ethanol (18). Mice in control group were kept without any stress application in the same housing conditions.

### Adeno-Associated Virus Vectors and Stereotaxic Injection

Mice received inhaled anesthesia with 2% isoflurane (RWD Life Science Co., LTD, Shenzhen, China) and positioned in a stereotaxic instrument (RWD Life Science Co., LTD). For knocking-down Clec2d in medial prefrontal cortex (mPFC), pAAV9-U6-mCherry-Clec2d-Mus-593 (titer: 4.25 × 10^12^, 5′→3′: GGATCAGCAGTACCAGGATCT; GenePharma Co., Ltd, Shanghai, China), or negative control virus pAAV9-U6-mCherry (titer: 1.04 × 10^13^, 5′→3′: TTCTCCGAACGTGTCACGT; GenePharma Co., Ltd) was injected into mPFC (AP, +1.70 mm; ML, ± 0.30 mm; DV, −2.80 mm; 600 nl for each hemisphere) by using a syringe (Hamilton Bonaduz AG, Switzerland) with micropipette (tip diameter, ~15 µm) attached to a KDS LEGATO 130 micropipette puller (RWD Life Science Co., LTD) at a flow rate of 0.1 µl/min followed by an additional 5 min to allow diffusion of the virus. Then, a standard single cannula (RWD Life Science Co., LTD) was bilaterally implanted in mPFC. Mice were housed for 3 weeks for recovery after adeno-associated virus (AAV) injection.

### Intracranial Microinjection

Recombinant nucleosomes (500 nl, 10 μg/ml; EpiCypher) or histones (500 nl, 5 μg/ml; New England BioLabs, Ipswich, MA, USA) were bilaterally delivered through implanted cannula, and then scarified for flow cytometry and fluorescence activated cell sorting (FACS) at the given time points. Recombinant nucleosomes and histones were diluted in artificial cerebrospinal fluid (ACSF) containing 150 mM NaCl, 10 mM d-glucose, 10 mM Hepes, 2.5 mM KCl, 1 mM MgCl_2_ (pH 7.35), 312 mOsm. For some experiments, nucleosomes were pretreated with DNase I (100 U; Sigma-Aldrich) to remove DNA or with activated protein C (APC; 100 nM; TopScience Co. Ltd., Shanghai, China) to cleave histones at 37°C for 1 h before intracranial microinjection.

### Open-Field Test

Mice were placed at the center of a cubic arena [50 cm (L) × 50 cm (W) × 40 cm (H)]. The free locomotion of mice traveled in 10 min was automatically monitored, recorded, and analyzed by Noldus EthoVision XT (Noldus Information Technology, Wageningen, Netherlands). The total distance and the percentage of time spent in the center area (center time% = center time/total time × 100%) were calculated.

### Elevated Plus Maze Test

Mice were placed at the center of a plus maze facing one of an open arm. The elevated plus maze (EPM) apparatus consisted of two opposing open arms [50 cm (L) × 10 cm (W)] and two opposing closed arms with roofless black walls (40-cm height with 60 cm elevated above the floor). The free locomotion of mice traveled in 10 min was automatically monitored, recorded, and analyzed by Noldus EthoVision XT (Noldus Information Technology). The percentage of time spent in the open arms [open arms time% = open arms time/(open arms time + closed arms time) × 100%] and the percentage of number of entries in the open arms [open arms entries % = open arms entries/(open arms entries + closed arms entries) × 100%] were calculated.

### Novel Object Recognition Test

Novel object recognition (NOR) test were consisted by habituation, training, and test section with a 60-min separated delay. Briefly, mice were placed at the center of a cubic arena [50 cm (L) × 50 cm (W) × 40 cm (H)] without any objects for habituation for 5 min. In the training session, two identical square woodblocks [2 cm (L) × 2 cm (W) × 2 cm (H)] were placed in opposite corners of the arena with enough space for mice to move past the woodblocks without interacting with them. In the test session, mice were presented with one identical woodblock (the familiar object) and another triangle woodblock [2 cm (L) × 1 cm (W) × 2 cm (H)] (the novel object). The investigations of mice sniffing and rearing on each object in 5 min were automatically monitored, recorded, and analyzed by Noldus EthoVision XT (Noldus Information Technology). The recognition index including the percentage of time spent with the novel object [recognition index (time) = time spent exploring novel object/time spent exploring both objects] and number of investigations [recognition index (investigations) = number of investigations for exploring novel object/number of investigations for exploring both objects] for exploring the novel object was calculated.

### Sucrose Preference Test

Mice were exposed to sterile water in two identical bottle on the training day 1 and to 1% sucrose (Sigma-Aldrich) solution on the training day 2. The location of two bottles was switched every 12 h to avoid location preference. In the test session, mice were exposed to one bottle of sterile water and the other identical bottle of 1% sucrose solution after 12-h water deprivation. The water and sucrose consumption for 24 h was recorded and the sucrose preference (SP) [SP (%) = sucrose consumption/(sucrose consumption + water consumption) × 100%] was calculated.

### Tail Suspension Test

Mice tails that were suspended to an acrylic bar (15-cm length with 30 cm elevated above the floor) in 6 min were automatically monitored, recorded, and analyzed by Noldus EthoVision XT (Noldus Information Technology). The immobility time in the last 5-min suspension period was recorded. The percentage of immobility time [immobility time% = immobility time/total time × 100%] was calculated.

### FS Test

Mice that were placed in a transparent cylindrical container (25-cm height with a diameter of 10 cm) filled with warm water (23°C~25°C) in 6 min were automatically monitored, recorded, and analyzed by Noldus EthoVision XT (Noldus Information Technology). The immobility time (floating behavior) in the last 5-min period was recorded. The percentage of immobility time [immobility time% = immobility time/total time × 100%] was calculated.

### Immunofluorescence Staining

Mice were deeply anesthetized with inhaling isoflurane (RWD Life Science Co., LTD) and perfused with 50 ml of 0.9% saline followed by 100 ml of 4% paraformaldehyde. Brain was removed and post-fixed in the same fixative overnight and then dehydrated for 48 h at 4°C in 30% sucrose solution. After being embedded with mounting medium (OCT, Tissue-Tek, Sakura, Torrance, CA, USA), transverse frozen sections (50-μm thick) were cut by using cryostat (CM1800; Leica, Heidelberg, Germany) and collected serially. The sections were rinsed in 0.01 M phosphate-buffered saline (PBS; pH 7.2~7.4) three times (10 min each) and blocked with 10% fetal bovine serum (FBS) in 0.01 M PBS containing 0.3% Triton X-100 for 30 min at room temperature (RT). And then, the sections were incubated 4 h at RT and then overnight at 4°C with rabbit anti-Iba-1 antibody (1:200; Abcam) and rat anti-CD68 antibody (1:200; Santa Cruz Biotechnology, Santa Cruz, CA, USA) or goat anti-Clec2d antibody (1:200; Abcam) and rabbit-anti-Eea1 (1:200; Abcam), rabbit-anti-Rab7 (1:200; Abcam), or rabbit-anti-calreticulin (1:200; Abcam). The sections were washed three times with 0.01 M PBS (10 min each) and then incubated for 2 h at RT with Alexa Fluor 488 donkey anti-rabbit or Alexa Fluor 488 donkey anti-goat and Alexa Fluor 594 donkey anti-rat immunoglobulin G (1:1000; Abcam). Finally, the sections were mounted with mounting medium containing 4′,6-diamidino-2-phenylindole (DAPI) (Abcam) after washing three times with 0.01 M PBS (10 min each) as well as obtained and three-dimensional reconstructed by using a confocal laser scan microscope (Zeiss LSM 980 Airyscan; Carl Zeiss Microscopy GmbH, Promenade, Jena, Germany).

### Flow Cytometry and FACS

Mice were sacrificed under deep anesthesia with inhaling isoflurane (RWD Life Science Co., LTD) and the brains were quickly removed in filtered FACS buffer containing 0.01 M PBS with 0.5% FBS on ice. Meninges, blood vessels, and choroid plexus were carefully separated and then bilateral mPFC was finely minced prior to digestion. Chopped mPFC was placed in glass homogenizer with a total volume of 1 ml of FACS buffer containing DNase I (20 U; Sigma-Aldrich), papain (20 U; Sigma-Aldrich), and dispase II (1.2 U; Sigma-Aldrich), and then processed with a mechanic dissociator at 6 rpm for 30 min at RT. Prior to use, papain was activated for 30 min at 37°C and 5% CO_2_. To stop all digestions, samples were diluted with cold FACS buffer and placed on ice. Solutions were then further homogenated and filtered through a 70-μm cell strainer (BD Biosciences, NJ, USA). The resulting single-cell suspension was centrifuged at 300*g* for 10 min at RT. Percoll (GE Healthcare, USA) gradient was used to remove myelin and enrich the homogenate for viable microglial cells. A stock solution of isotonic Percoll was prepared (9:1 Percoll in 0.1 M PBS). Cell pellets were resuspended in a 30% stock isotonic Percoll (SIP) diluted with HyClone Dulbecco’s modified Eagle’s medium (Gibco, Thermo Fisher Scientific, Waltham, MA, USA). Gradient were centrifuged without brake for at 300*g* for 30 min at RT. After centrifugation, cells were collected and washed with cold FACS buffer, centrifugated for 10 min at 300*g*, and resuspended and incubated in 0.5 ml of 1× red blood cell lysis buffer (Solarbio Life Science) at RT for 5 min. Solutions were centrifuged at 300*g* for 10 min and cells were finally resuspended in 100 μl of FACS buffer on ice before flow cytometry staining. Fc receptor blocking reagent for mouse (1:100; BioLegend, USA) was added to the FACS buffer containing the cell suspension to block surface antigens in microglial cells or macrophages and incubated for 10 min. For surface staining, rabbit anti-Clec2d antibody (1:200; Abcam) was incubated for 30 min, washed with cold FACS buffer, and centrifugated for 10 min at 300*g*; then, a mixture containing fluorochrome-conjugated antibodies recognizing mouse CD45-PE, CD11b-FITC, I-A/I-E-BV421 (1:200; BD Biosciences), and Alexa Fluor 594 donkey anti-rabbit (1:1000; Abcam) was incubated for another 30 min. Samples were washed with 500 μl of FACS buffer and centrifugated for 10 min at 300*g* in each step. For intracellular staining, samples were incubated with 250 μl of 1 × fixation/permeabilization solution (BD Biosciences) for 20 min at RT after surface staining. And then, samples were washed with 500 μl of 1× BD Perm/Wash™ buffer (BD Biosciences) and centrifugated for 10 min at 300*g*. Cells were resuspended with rabbit anti-Clec2d antibody (1:200; Abcam), incubated for 30 min, washed with cold FACS buffer, centrifugated for 10 min at 300*g*, and then incubated with Alexa Fluor 594 donkey anti-rabbit (1:1000; Abcam) for another 30 min. For ROS incubation, cell pellets were incubated with 2',7'-Dichlorodihydrofluorescein diacetate (DCFH-DA) (1:1000; Beyotime Biotechnology, Shanghai, China) for 20 min at 37°C and 5% CO_2_ before surface staining. Finally, cell pellets were resuspended in 250 μl of FACS buffer and samples were immediately used for flow cytometry by using Beckman Cytoflex LX (Beckman Coulter, Inc., CA, USA) or FACS by using MoFlo Astrios EQS Cell Sorter (Beckman Coulter, Inc.).

### Western Blotting

CD11b^+^CD45^Low^ microglia were collected by FACS with a number of 1 × 10^5^. Cell pellets were lysed with 2× loading buffer with proteinase and phosphatase inhibitor cocktail (Solarbio Life Science). The electrophoresis samples were heated at 100°C for 10 min and loaded onto 10% SDS–polyacrylamide gels with standard Laemmli solutions (Bio-Rad Laboratories, CA, USA). After the proteins were electroblotted onto a polyvinylidene difluoride membrane (Immobilon-P, Millipore, Billerica, MA, USA), the membranes were placed in a blocking solution containing tris-buffered saline with 0.02% Tween 20 (TBS-T; Sigma-Aldrich) and 5% non-fat dry milk (Sigma-Aldrich) and incubated for 60 min under gentle agitation at RT, then at 4°C for overnight with goat anti-Clec2d antibody (1:1000; Abcam), rabbit anti-TLR9 antibody (1:1000; Abcam), rabbit anti-citH3 antibody (1:1000; Abcam), goat anti-citrullinating enzyme peptidylarginine deiminase type 4 (PAD4) antibody (1:1000; Abcam), rabbit anti–nuclear factor-κB (NF-κB) p65 antibody (1:1000; Cell Signaling Technology Inc.), rabbit anti–p-NF-κB p65 antibody (1:1000; Cell Signaling Technology Inc.), rabbit anti-inhibitor of NF-κB α (IκBα) antibody (1:1000; Cell Signaling Technology Inc.), rabbit anti–p-IκBα antibody (1:1000; Cell Signaling Technology Inc.), rabbit anti–NOD-like receptors (NLRs) family pyrin domain-containing 3 (NLRP3) antibody (1:1000; Cell Signaling Technology Inc.), rabbit anti-cleaved caspase-1 antibody (1:1000; Cell Signaling Technology Inc.), rabbit anti–IL-1β antibody (1:1000; Cell Signaling Technology, Inc.), rabbit anti-cleaved IL-1β antibody (1:1000; Cell Signaling Technology, Inc.), or mouse anti–β-actin antibody (1:5000; Sigma-Aldrich). Bound primary antibodies were detected by incubating with anti-mouse, anti-rabbit, or anti-goat horseradish peroxidase–conjugated secondary antibody (1:10000; Amersham Pharmacia Biotech Inc., Piscataway, NJ, USA) for 2 h under gentle agitation at RT. Between each step, the immunoblots were rinsed with TBS-T three times (10 min each). Protein blots’ densities were detected by using enhanced chemiluminescence (Solarbio Life Science) and analyzed in the Bio-Rad ChemiDoc Imaging System (Bio-Rad Laboratories Ltd, USA). Immunoreactive bands were quantified and normalized to β-actin.

### Enzyme Linked Immunosorbent Assay

CORT, IL-1β, and extracellular nucleosomes levels were measured by using enzyme linked immunosorbent assay (ELISA) kits for CORT (Cloud Clone Crop., Wuhan, China), IL-1β (Cloud Clone Crop.), or nucleosomes (Cell Death Detection ELISA^PLUS^, Roche, Hvidovre, Denmark) according to the instructions.

### Statistical Analysis

Data were expressed as mean ± standard error of the mean (SEM) unless otherwise specified. Paired or independent Student’s *t*-test was used for two groups comparison and two- or three-way or repeated measurement analysis of variance (ANOVA) followed by Tukey’s *post hoc* test was used for multiple comparison as appropriate. The area under the time-course curves values during the analysis time was used to measure the summed effects of body weight. The linear regression was conducted to analyze the correlation between nucleosomes in CSF and IL-1β in mPFC. Sholl analysis was applied to analyze microglial morphology by counting the number and length of branch tips with ImageJ (http://imagej.net). All these data were analyzed by using GraphPad Prism version 8.3.0 for Windows (Graph Pad Software, San Diego California USA, www.graphpad.com). *P* < 0.05 was considered as statistical significance.

## Results

### Mice With Chronic Stress Exhibited Negative Effects on Daily Weight Gain and Emotional Behaviors

**Figure 1:**
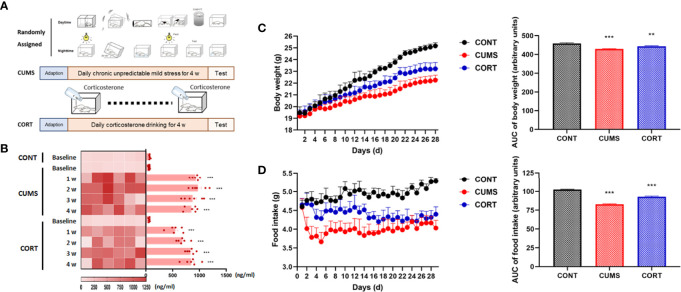
Mice with chronic stress exhibited negative effects on daily weight gain and stress. **(A)** Mice in chronic stress received CUMS or CORT drinking for 4 weeks. **(B)** Serum CORT level was measured at baseline, 1, 2, 3, and 4 weeks after receiving chronic stress (repeated measurement ANOVA with Tukey’s *post hoc* test, ***P < 0.001, compared with baseline; n = 6). **(C)** Daily body weight was recorded and area under the time-course curve (AUC) was analyzed (two-way ANOVA with Tukey’s *post hoc* test, **P < 0.01, ***P < 0.001, compared with CONT; n = 12). **(D)** Daily food intake was recorded and AUC was analyzed (two-way ANOVA with Tukey’s *post hoc* test, ***P < 0.001, compared with CONT; n = 12).

Mice with chronic stress were experienced CUMS and CORT drinking for 4 weeks (**Figure 1A**). Compared with CONT group, mice in CUMS and CORT groups showed substantially boosted serum CORT (*P* < 0.001; **Figure 1B**), indicating a stressful condition of mice during chronic stress. Besides, mice in CUMS and CORT groups also presented negative effects on daily weight gain, compared with CONT group, indicated by significantly reduced body weight (*P* < 0.001; **Figure 1C**) and daily food intake (*P* < 0.001; **Figure 1D**).

**Figure 2:**
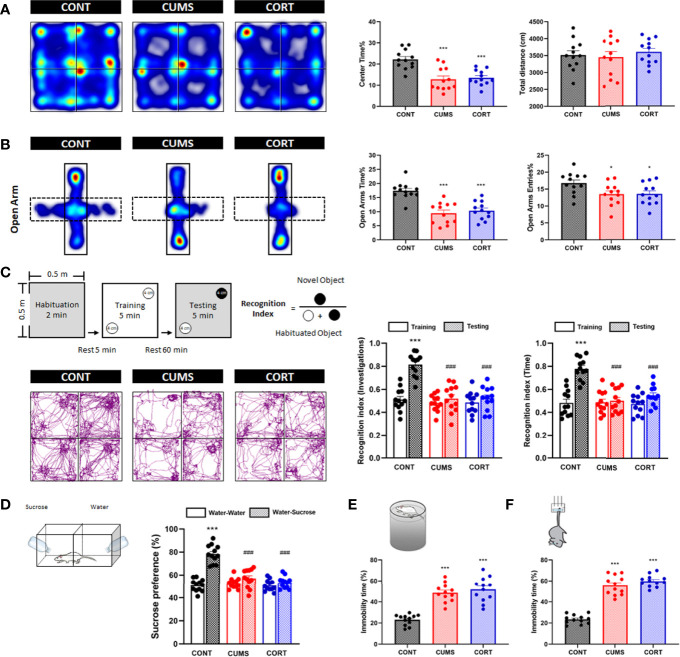
Mice with chronic stress exhibited negative emotional behaviors. **(A)** Representative heat maps for CONT, CUMS, and CORT groups in OF test were presented, and center time% and total distance were analyzed (two-way ANOVA with Tukey’s *post hoc* test, ***P < 0.001, compared with CONT; n = 12). **(B)** Representative heat maps for CONT, CUMS, and CORT groups in EPM test were presented, and open arm time% and open arm entries% were analyzed (two-way ANOVA with Tukey’s *post hoc* test, *P < 0.05, ***P < 0.001, compared with CONT; n = 12). **(C)** Experimental protocol and representative trajectory images for CONT, CUMS, and CORT groups in NOR test were presented, and recognition index of investigations and time was analyzed (paired Student’s *t*-test, ***P < 0.001, compared with training. Two-way ANOVA with Tukey’s *post hoc* test, ### *P* < 0.001, compared with CONT-testing; n = 12). **(D)** Experimental protocol for SP test was presented, and sucrose preference was analyzed (paired Student’s *t*-test, ***P < 0.001, compared with water–water. Two-way ANOVA with Tukey’s *post hoc* test, ###*P* < 0.001, compared with CONT–water–sucrose; n = 12). **(E)** Experimental protocol for FS test was presented, and immobility time% was analyzed (two-way ANOVA with Tukey’s *post hoc* test, ***P < 0.001, compared with CONT; n = 12). **(F)** Experimental protocol for TS test was presented, and immobility time% was analyzed (Two-way ANOVA with Tukey’s *post hoc* test*post hoc*, ***P < 0.001, compared with CONT; n = 12).

Chronic stress was associated with negative emotional behaviors. Compared with CONT group, mice in CUMS and CORT groups presented a significantly decreased center time% (*P* < 0.001) with similar total distance in open-field (OF) test (*P* > 0.05; **Figure 2A**), as well as a reduced OA time% (*P* < 0.001) and open arms (OA) entries% in EPM test (*P* < 0.05; **Figure 2B**), indicating the anxiety-like emotion without locomotion impairment. Compared with CONT group, mice in CUMS and CORT groups presented a significantly decreased recognition index on novel object investigations and time in NOR test (*P* < 0.001; **Figure 2C**), indicating the cognitive dysfunction. Compared with CONT group, mice in CUMS and CORT groups presented a significantly decreased SP in SP test (*P* < 0.001; **Figure 2D**), as well as a prolonged immobility time% in both FS (*P* < 0.001; **Figure 2E**) and tail suspension (TS) tests (*P* < 0.001; **Figure 2F**), indicating the depression-like emotion.

### Extracellular Nucleosomes Were Dictated in mPFC of Mice With Chronic Stress

**Figure 3:**
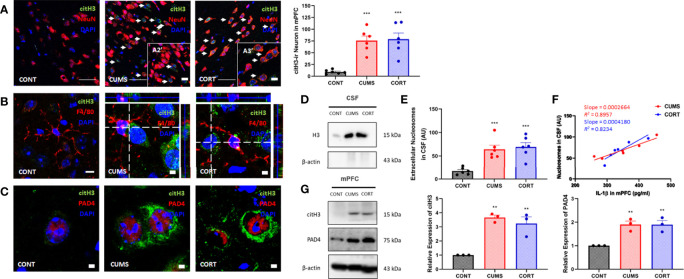
Chronic stress induced extracellular histones release and extracellular trap formation in mPFC. **(A)** Representative immunofluorescence images showing the citH3^+^ and NeuN^+^ in CONT, CUMS, and CORT groups were presented, and citH3-ir neurons in mPFC was analyzed (two-way ANOVA with Tukey’s *post hoc* test*post hoc*, ***P < 0.001, compared with CONT. Two replica for n = 3). Scale bars: 20× and, 40×. **(B)** Representative immunofluorescence images showing the relationship of F4/80^+^ microglia and citH3^+^ cells in CONT, CUMS, and CORT groups were presented. Scale bar: 126×. **(C)** Representative immunofluorescence images showing the relationship of citH3 and PAD4 in CONT, CUMS, and CORT groups were presented. Scale bar: 126×. **(D)** Representative Western blots showing extracellular histone H3 in CSF. **(E)** Concentration of nucleosomes in CSF was analyzed (two-way ANOVA with Tukey’s *post hoc* test*post hoc*, ***P < 0.001, compared with CONT; n = 6). **(F)** Regression analysis between nucleosomes in CSF and IL-1β in mPFC was presented (linear regression; n = 6). **(G)** Representative Western blots showing citH3 and PAD4 in mPFC were presented. Relative expression of citH3 and PAD4 was analyzed (two-way ANOVA with Tukey’s *post hoc* test*post hoc*, **P < 0.01, compared with CONT; n = 3). *post hoc*.

Chronic stress induces cell death and necrotic cells release DAMPs and triggered neuroinflammation (19, 20). Intracellular histones release predominantly depends on posttranscriptional modification that histones citrullination mediated by their encoding PAD4 (21). Our findings suggested that the expression of citrullinated histones H3 and PAD4 was significantly increased (*P* < 0.01; **Figures 3A,G**) and showed colocalization (**Figure 3C**). Moreover, citH3^+^ neurons showed a close relationship with microglia (**Figure 3B**) in mPFC in CUMS and CORT groups, compared with CONT group. We further confirmed extracellular histones (**Figure 3D**) and extracellular nucleosomes in CSF in CUMS and CORT groups (*P* < 0.05, **Figure 3E**). Importantly, positive correlations were found in nucleosome in CSF and IL-1β in mPFC in CUMS and CORT group with *r^2^* = 0.8957 and 0.8234, respectively (**Figure 3F**).

### Mice With Chronic Stress Were Accompanied With Microglial Activation and Inflammation in mPFC

**Figure 4:**
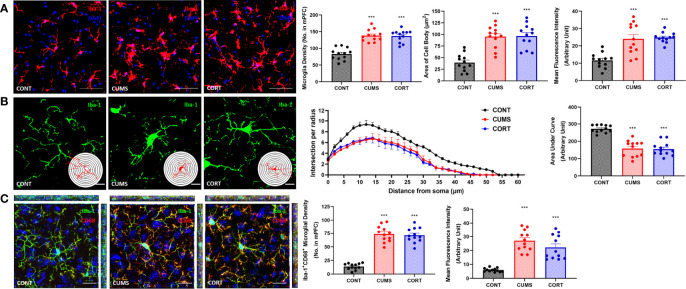
Mice with chronic stress were accompanied with microglial activation in mPFC. **(A)** Representative immunofluorescence images showing the Iba-1^+^ microglia in CONT, CUMS, and CORT groups were presented, and the microglial density, area of cell body, and the MFI of Iba-1 were analyzed (two-way ANOVA with Tukey’s *post hoc* test*post hoc*, ***P < 0.001, compared with CONT. Two replica for n = 6). Scale bar: 40×. **(B)** Representative immunofluorescence images showing the 3D reconstruction of Iba-1^+^ microglia in CONT, CUMS, and CORT groups were presented, and Sholl analysis and their AUCs were analyzed (two-way ANOVA with Tukey’s *post hoc* test*post hoc*, ***P < 0.001, compared with CONT. Two replica for n = 6). Scale bar: 126×. **(C)** Representative immunofluorescence images showing the co-localization of CD68^+^ and Iba-1^+^ microglia in CONT, CUMS, and CORT group were presented, and the Iba-1^+^CD68^+^ microglial density and also the MFI of CD68 were analyzed (two-way ANOVA with Tukey’s *post hoc* test*post hoc*, ***P < 0.001, compared with CONT. Two replica for n = 6). Scale bar: 63×.

Increasing evidence has suggested that neuroinflammation is contributed to emotional disorders induced by chronic stress, and microglia play the executive role of immunity and inflammation in CNS. Therefore, we primarily focused on microglial biological function during chronic stress. Our morphological results and Sholl analysis showed that microglial pro-inflammatory activation in CUMS and CORT groups was indicated by proliferated microglia (*P* < 0.001; **Figure 4A**) with increased soma volume and retracted processes (*P* < 0.001; **Figure 4B**). Moreover, we investigated CD68 expression, a classic phagocytic marker for activated monocytes and macrophages, in microglia. The results indicated that increased CD68 expression was observed in mPFC of mice with CUMS and CORT, compared with CONT group (*P* < 0.001; **Figure 4C**), indicating a pro-phagocytic activation state of microglia in mPFC. Since microglial inflammation was associated with neuronal synaptic plasticity, we additionally confirmed the significantly decreased density of spine, specifically for mushroom type, in mPFC of mice with CUMS and CORT, compared with CONT group by using sparse labeling technique (**Supplementary Figure 1**).

**Figure 5:**
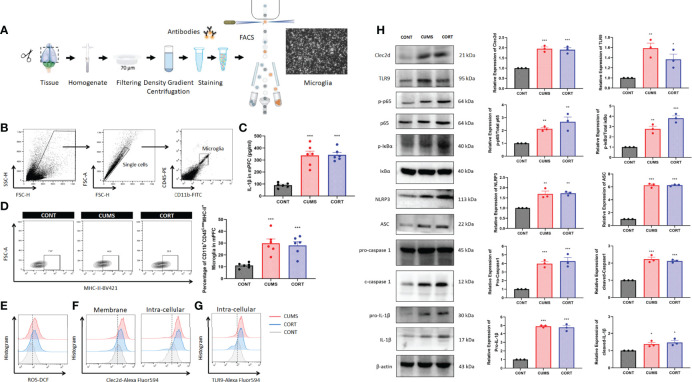
Chronic stress induced microglial activation in mPFC. **(A)** Experimental protocol for FACS was presented. **(B)** Gate strategy for CD11b^+^45^Low^ microglia was presented. **(C)** Concentration of IL-1β in mPFC was measured (two-way ANOVA with Tukey’s *post hoc* test*post hoc*, ***P < 0.001, compared with CONT; n = 6). **(D)** Representative flow cytometry images showing the percentage of CD11b^+^CD45^Low^MHC-II^+^ microglia in mPFC of CONT, CUMS, and CORT groups, as well as the statistical analysis (two-way ANOVA with Tukey’s *post hoc* test*post hoc*, ***P < 0.001, compared with CONT; n = 6) were presented. **(E)** Representative histogram images showing CD11b^+^CD45^Low^ microglial ROS in mPFC were presented. **(F)** Representative histogram images showing CD11b^+^CD45^Low^ microglial Clec2d on the membrane and intracellular in mPFC were presented. **(G)** Representative histogram images showing CD11b^+^CD45^Low^ microglial intracellular TLR9 in mPFC were presented. **(H)** Representative Western blots showing microglial Clec2d, TLR9, NF-κB pathway, and NLRP3 inflammasome were presented. Relative expression of Clec2d, TLR9, p-p65, p-IκBα, NLRP3, ASC, pro-caspase-1, cleaved caspase-1, pro-IL-1β, and cleaved IL-1β was analyzed (two-way ANOVA with Tukey’s *post hoc* test*post hoc*, *P < 0.05, **P < 0.01,***P < 0.001, compared with CONT; n = 3).

For specifically identifying more the change of microglia during chronic stress, we further investigated by using flow cytometry and cell sorting (**Figure 5A**). Microglia in mPFC were harvested and Western blot was performed. The results from CD11b^+^CD45^low^MHC-II^+^ microglia in mPFC (**Figure 5B**) showed that a significant higher level of cerebral IL-1β (*P* < 0.001, **Figure 5C**) and a proportion of activated microglia (*P* < 0.001, **Figure 5D**) with increased ROS production (**Figure 5E**) were shown in CUMS and CORT groups, compared with CONT group. Moreover, the Western blot data also conformed the primed NF-κB signaling pathway indicated by upregulated p-p65, p-IκBα, pro-caspase 1, and pro–IL-1β, as well activated NLRP3 inflammasome indicated by upregulated NLRP3, ASC, cleaved-caspase 1, and cleaved IL-1β, in CUMS and CORT groups, compared with CONT group (*P* < 0.05; **Figure 5H**). Besides, microglial Clec2d, a newly defined cellular death sensor, and TLR9, a classic intracellular DNA sensor, were also found to be increased in CUMS and CORT groups, compared with CONT group (**Figures 5F-H**).

### Extracellular Nucleosomes Accelerated Microglial Oxidative Stress and Pro-Inflammatory Activation But Not Histones in mPFC

**Figure 6:**
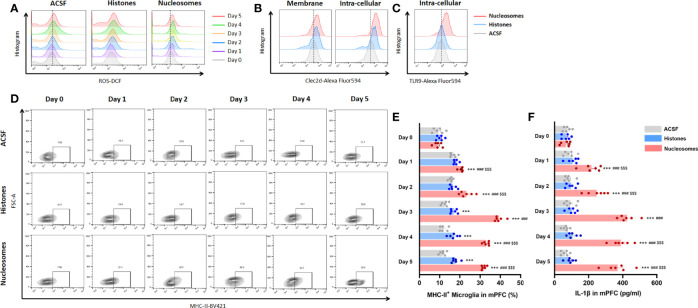
Extracellular nucleosomes accelerated microglial oxidative stress and pro-inflammatory activation but not histones in mPFC. **(A)** Representative histogram images showing CD11b^+^CD45^Low^ microglial ROS before and 1~5 days after ACSF, histones, and nucleosomes stereotactic injection in mPFC were presented. **(B)** Representative histogram images showing CD11b^+^CD45^Low^ microglial Clec2d on the membrane and intracellular on day 3 after ACSF, histones, and nucleosomes stereotactic injection in mPFC were presented. **(C)** Representative histogram images showing intracellular TLR9 on day 3 after ACSF, histones, and nucleosomes stereotactic injection in mPFC were presented. **(D)** Representative flow cytometry images showing the percentage of CD11b^+^CD45^Low^MHC-II^+^ microglia before and 1~5 days after ACSF, histones, and nucleosomes stereotactic injection in mPFC were presented. **(E)** Percentage of CD11b^+^CD45^Low^MHC-II^+^ microglia before and 1~5 days after ACSF, histones, and nucleosomes stereotactic injection in mPFC was analyzed (repeated measurement ANOVA with Tukey’s *post hoc* test*post hoc*, ***P < 0.001, compared with CONT; ###*P* < 0.001, compared with Histones; $$$*P* < 0.001, compared with nucleosomes day 0; n = 6). **(F)** Concentration of IL-1β before and 1~5 days after ACSF, histones, and nucleosomes stereotactic injection in mPFC was analyzed (repeated measurement ANOVA with Tukey’s *post hoc* test*post hoc*, ***P < 0.001, compared with CONT; ###*P* < 0.001, compared with histones; $$$ *P* < 0.001, compared with nucleosomes day 0; n = 6).

For specifically identifying the pro-inflammatory role and mechanism of extracellular nucleosomes and histones, stereotactic injection of recombinant nucleosomes and histones was conducted in mPFC *in vivo*. Previous studies indicated that injection of 1.25 mg of purified histones was lethal within 1 h, and serum levels of histones as high as 3 ng/ml presented coagulopathy, endothelial damage, and organ dysfunction in human (22, 23). Moreover, a concentration of 500 μg/ml of externalized histones H4 orchestrated chronic inflammation by inducing lytic smooth muscle cells death and 200 nM extracellular histones H1 exhibited significant neurotoxicity (24, 25). However, whether extracellular nucleosomes exhibited cytotoxic effects remained controversial. Some studies reported that isolated nucleosomes do not induce cell death of cultured endothelial cells *in vitro* (26, 27). Nonetheless, some have described that nucleosomes induced necrotic cell death specifically in cultured lymphocytes and *in vivo* in a dose- and time-dependent manner (28). Thus, we investigated the concentration-dependent promicroglial death and promicroglial activation profile for recombinant nucleosomes and histones prior to *in vivo* study. The results showed a concentration of more than 10 μg/ml histones of the presented significant neurotoxicity for primary neuron, but not for nucleosomes, even at a relatively high concentration of 15 μg/ml (**Supplementary Figure 2**). Considering that cytotoxicity triggers chronic inflammation and microglial activation secondary to cell death, we performed bilateral stereotactic injection of 10 μg/ml of recombinant nucleosomes and 5 μg/ml histones in mPFC in a volume of 1 μl. Interestingly, nucleosomes induced significantly microglial oxidative stress and pro-inflammatory activation indicated by more ROS production, upregulated of major histocompatibility complex II (MHC-II) expression, and IL-1β secretion in a time-dependent manner, while the pro-inflammatory profile was limited for histones injection (*P* < 0.001, **Figures 6A, D–F**).

### Extracellular Nucleosomes Accelerated Microglial Inflammation *via* Clec2d and TLR9

**Figure 7:**
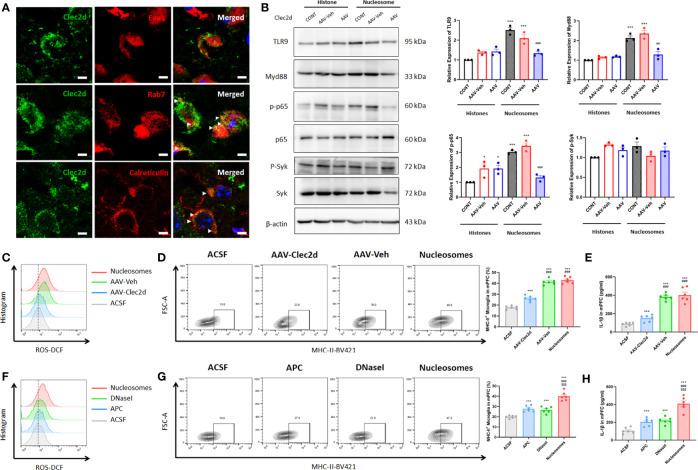
Extracellular nucleosomes accelerated microglial inflammation *via* Clec2d and TLR9. **(A)** Representative immunofluorescence images showing the subcellular location of Clec2d and Eea1, Rab7, and calreticulin, as well as their relationship in primary microglia were presented. Scale bar: 150×. **(B)** Representative Western blots showing the expression of microglial TLR9, Myd88, p-p65, and p-Syk on 3 days after ACSF, histones, or nucleosomes stereotactic injection in mPFC with Clec2d knocking-down were presented. Relative expression of TLR9, Myd88, p-p65, and p-Syk on 3 days after ACSF, histones, or nucleosomes stereotactic injection in mPFC with Clec2d knocking-down was analyzed (two-way ANOVA with Tukey’s *post hoc* test*post hoc*, *P < 0.05, ***P < 0.001, compared with histones; ##*P* < 0.01, ###*P* < 0.001, compared with Nucleosomes-AAV-Veh; n = 3). **(C)** Representative flow cytometry images showing CD11b^+^CD45^Low^ microglial ROS on 3 days after ACSF or nucleosomes stereotactic injection in mPFC with Clec2d knocking-down were presented. **(D)** Representative flow cytometry images and statistical analysis showing the percentage of CD11b^+^CD45^Low^MHC-II^+^ microglia on 3 days after ACSF or nucleosomes stereotactic injection in mPFC with Clec2d knocking-down were presented (two-way ANOVA with Tukey’s *post hoc* test*post hoc*, ***P < 0.001, compared with ACSF; ###*P* < 0.001, compared with AAV-Clec2d; n = 6). **(E)** Concentration of IL-1β on 3 days after ACSF or nucleosomes stereotactic injection in mPFC with Clec2d knocking-down was analyzed (two-way ANOVA with Tukey’s *post hoc* test*post hoc*, ***P < 0.001, compared with ACSF; ###*P* < 0.001, compared with AAV-Clec2d; n = 6). **(F)** Representative flow cytometry images showing CD11b^+^CD45^Low^ microglial ROS on 3 days after ACSF, APC pretreatment, DNase I pretreatment, or nucleosomes stereotactic injection in mPFC were presented. **(G)** Representative flow cytometry images and statistical analysis showing the percentage of CD11b^+^CD45^Low^MHC-II^+^ microglia on 3 days after ACSF, APC pretreatment, DNase I pretreatment, or nucleosomes stereotactic injection in mPFC were presented (two-way ANOVA with Tukey’s *post hoc* test*post hoc*, ***P < 0.001, compared with ACSF; $$$*P* < 0.001, compared with APC; $$$*P* < 0.001, compared with DNase I; n = 6). **(H)** Concentration of IL-1βon 3 days after ACSF, APC pretreatment, DNase I pretreatment, or nucleosomes stereotactic injection in mPFC was analyzed (two-way ANOVA with Tukey’s *post hoc* test*post hoc*, ***P < 0.001, compared with ACSF; $$$*P* < 0.001, compared with APC; $$$*P* < 0.001, compared with DNase I; n = 6).

Considering that the microglial inflammation reached the peak on 3 days after stereotactic injection, we further investigated Clec2d and TLR9 expression on 3 days after stereotactic injection. The results revealed that Clec2d on the member and intracellular was significantly increased, while the significant increase of TLR9 was shown in nucleosomes injection, instead of histones (**Figures 6B, C**). Furthermore, the results of Clec2d intracellular expression showed that Clec2d was colocalized with late endosome (indicated by colocalization of Clec2d and Rab7) and endoplasmic reticulum (indicated by colocalization of Clec2d and calreticulin), instead of early endosome (**Figure 7A**). Together with the results that significant increase of intracellular Clec2d, this might indicate an internalization of Clec2d with nucleosomes and histones stimulation. Considering the absence of enlarged microglial inflammation in histones injection, we further investigated microglia intracellular pro-inflammatory signaling pathway in mPFC on 3 days after nucleosomes and histones stereotactic injection with or without Clec2d knocking-down, respectively. Compared with histones stereotactic injection, nucleosomes significantly upregulated TLR9, Myd88, and p-p65 expression, while, previously, local knocking-down of Clec2d by AAV-Clec2d obviously eliminated the activation of TLR9–NF-κB signaling pathway (*P* < 0.01, **Figure 7B**). However, no significant activation of the other mitogen-activated protein kinase (MAPK) pathways, including p-ERK, p-p38, and p-Jnk, was detected (**Supplementary Figure 3**). Considering the previous study has revealed that Clec2d was not a signaling receptor, and together with the fact that stimulation of Clec2d with histones did not induce cytokine responses (16), we further investigated the Syk signaling pathway, the knowing downstream signaling kinase of activating C-type lectin receptors (CLRs). The results showed no obvious detectable Clec2d-dependent activation of Syk with nucleosomes and histones stimulation (*P* > 0.05, **Figure 7B**). Importantly, knocking-down of Clec2d by AAV-Clec2d inhibited microglial oxidative stress, pro-inflammatory activation, and cytokine production induced by extracellular nucleosomes (*P* < 0.001, **Figures 7C-E**). Moreover, the ability of extracellular nucleosomes to stimulate ROS production, MHC-II expression, and IL-1β secretion in microglia would be abolished when it was pretreated with DNase I to remove DNA or APC to cleave histones (*P* < 0.001, **Figures 7F-H**). For further identifying the role of TLR9, we investigated the microglial inflammation induced by recombinant nucleosomes with Clec2d or/combined with TLR9 antagonist E6446 dihydrochloride treatment. The results showed that both Clec2d knocking-down, TLR9 inhibitor, and Clec2d knocking-down combined with TLR9 inhibitor significantly inhibited microglial IL-1β expression. Moreover, Clec2d knocking-down combined with TLR9 inhibitor was superior to Clec2d knocking-down, while no obvious synergic effect was found when compared with TLR9 alone (**Supplementary Figure 4**). Taken together, our results revealed that the amplified pro-inflammatory mechanism of nucleosomes (histones-bound DNA) might be derived from Clec2d-mediating histones-bound DNA internalization and DNA-mediating endosomal TLR9-dependent activation of NF-κB signaling pathway.

### Clec2d Knocking-Down Reduced Microglial Activation and Inflammation in mPFC As Well As Improved Negative Emotional Behaviors

**Figure 8:**
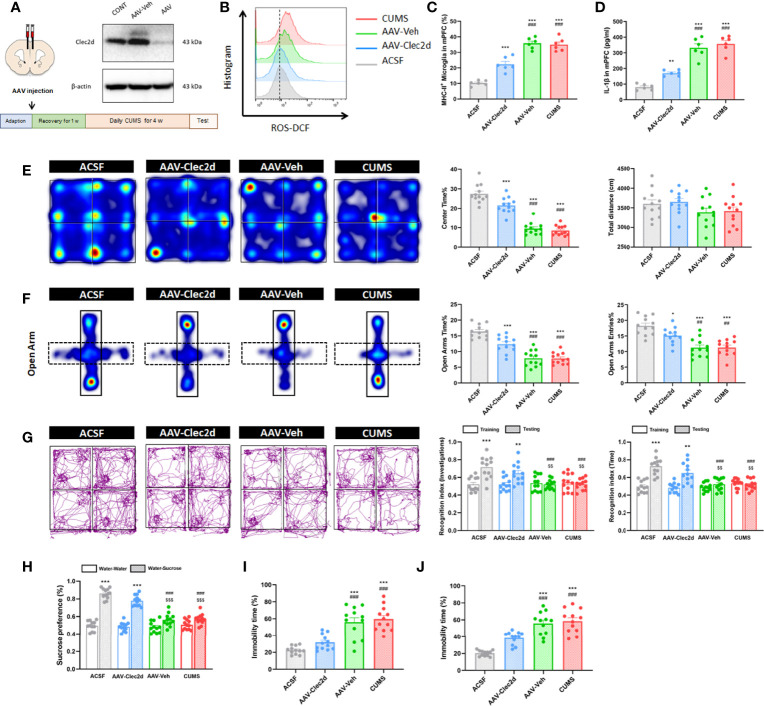
Clec2d knocking-down reduced microglial activation and inflammation in mPFC and improved negative emotional behaviors in mice with CUMS. **(A)** Experimental protocol for mice receiving CUMS with Clec2d knocking-down in mPFC and representative Western blots showing the molecular expression were presented. **(B)** Representative histogram images showing CD11b^+^CD45^Low^ microglial ROS in mice receiving CUMS with Clec2d knocking-down in mPFC were presented. **(C)** The percentage of CD11b^+^CD45^Low^MHC-II^+^ microglia in mice receiving CUMS with Clec2d knocking-down in mPFC was analyzed (two-way ANOVA with Tukey’s *post hoc* test*post hoc*, ***P < 0.001, compared with ACSF; ###*P* < 0.001, compared with AAV-Clec2d; n = 6). **(D)** Concentration of IL-1β in mice receiving CUMS with Clec2d knocking-down in mPFC was analyzed (two-way ANOVA with Tukey’s *post hoc* test*post hoc*, **P < 0.01, ***P < 0.001, compared with ACSF; ###*P* < 0.001, compared with AAV-Clec2d; n = 6). **(E)** Representative heat maps for ACSF, AAV-Clec2d, AAV-Veh, and CUMS group in OF test were presented, and center time% and total distance were analyzed (two-way ANOVA with Tukey’s *post hoc* test*post hoc*, ***P < 0.001, compared with ACSF; ###*P* < 0.001, compared with AAV-Clec2d; n = 12). **(F)** Representative heat maps for ACSF, AAV-Clec2d, AAV-Veh, and CUMS group in EPM test were presented, and open arm time% and open arm entries% were analyzed (two-way ANOVA with Tukey’s *post hoc* test*post hoc*, *P < 0.05, ***P < 0.001, compared with ACSF; ##*P* < 0.01, ###*P* < 0.001, compared with AAV-Clec2d; n = 12). **(G)** Representative trajectory images for ACSF, AAV-Clec2d, AAV-Veh, and CUMS group in NOR test were presented, and recognition index of investigations and time was analyzed (paired Student’s *t*-test, **P < 0.01, ***P < 0.001, compared with training. Two-way ANOVA with Tukey’s *post hoc* test*post hoc*, ###*P* < 0.001, compared with ACSF-testing; $$*P* < 0.01, compared with AAV-Clec2d; n = 12). **(H)** Sucrose preference was analyzed (paired Student’s *t*-test, ***P < 0.001, compared with water–water. Two-way ANOVA with Tukey’s *post hoc* test*post hoc*, ###*P* < 0.001, compared with ACSF-water-sucrose; $$$*P* < 0.001, compared with AAV-Clec2d; n = 12). **(I)** Immobility time% in FS test was analyzed (two-way ANOVA with Tukey’s *post hoc* test*post hoc*, ***P < 0.001, compared with ACSF; ###*P* < 0.001, compared with AAV-Clec2d; n = 12). **(J)** Immobility time% in TS test was analyzed (two-way ANOVA with Tukey’s *post hoc* test*post hoc*, ***P < 0.001, compared with ACSF; ###*P* < 0.001, compared with AAV-Clec2d; n = 12).

For further confirming the role of Clec2d-dependent extracellular nucleosomes in mPFC of mice with chronic stress, bilateral stereotactic injection of AAV-Clec2d was performed to knock down Clec2d in mPFC prior to receiving CUMS and CORT drinking (**Figure 8A** and **Supplementary Figure 5**). Our results revealed an obvious decrease of microglial ROS production (**Figure 8B** and **Supplementary Figure 5**), MHC-II expression (*P* < 0.001, **Figure 8C** and **Supplementary Figure 5**), and IL-1β secretion (*P* < 0.001, **Figure 8D** and **Supplementary Figure 5**) in mPFC. Moreover, Clec2d knocking-down also significantly rescued the decreased density of mushroom type spine in mPFC of mice with CUMS and CORT (**Supplementary Figure 6**). Notably, the negative emotional behaviors were significantly improved indicated by increased center time% in OF (*P* < 0.001, **Figure 8E** and **Supplementary Figure 5**), OA time% and OA entries% in EPM (*P* < 0.01, **Figure 8F** and **Supplementary Figure 5**), recognition index in NOR (*P* < 0.01, **Figure 8G** and **Supplementary Figure 5**), sucrose preference in SP (*P* < 0.001, **Figure 8H** and **Supplementary Figure 5**), and immobility time% in both FS (*P* < 0.001, **Figure 8I** and **Supplementary Figure 5**) and TS (*P* < 0.001, **Figure 8J** and **Supplementary Figure 5**) tests in AAV-Clec2d group, compared with CUMS and AAV-Veh groups.

## Discussion

**Figure 9:**
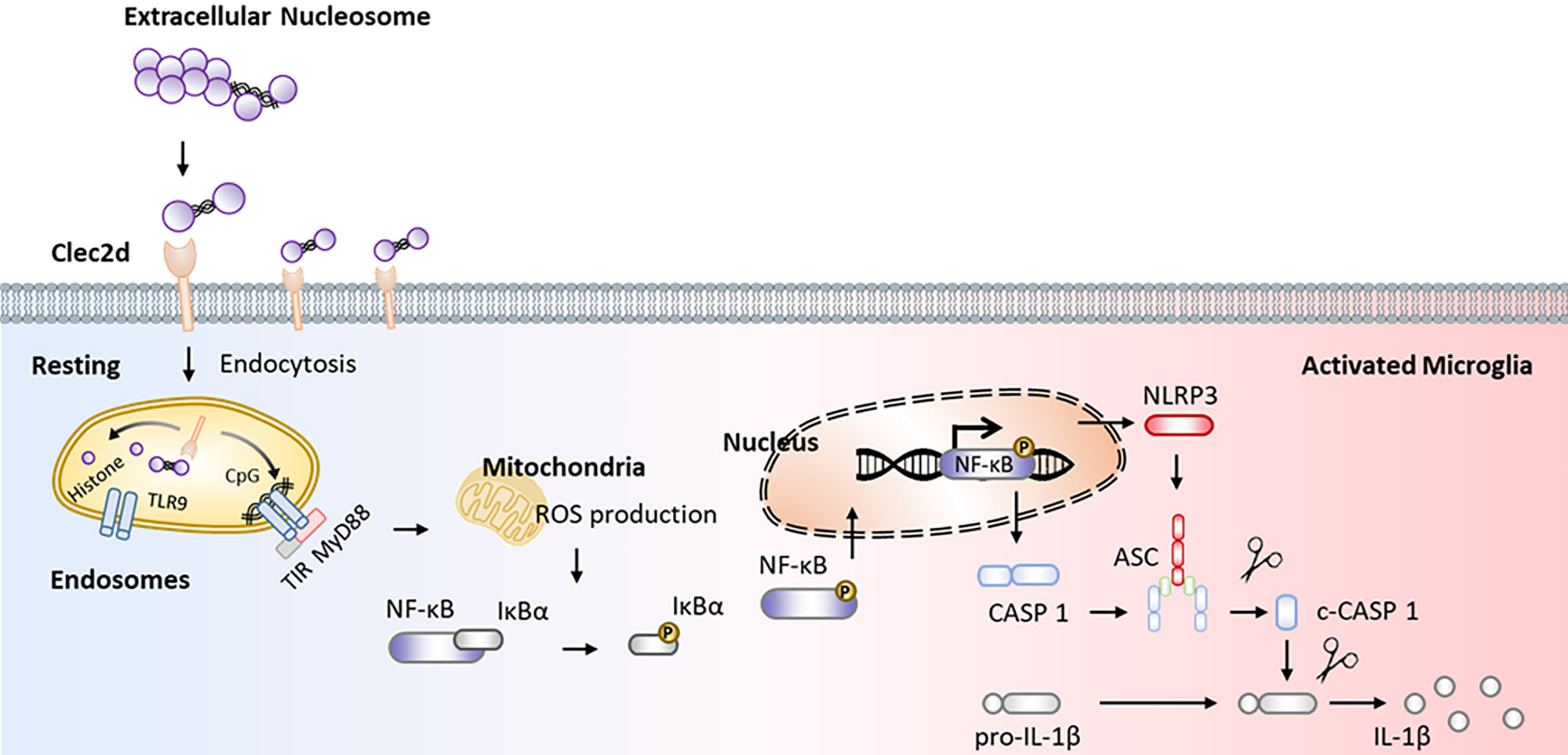
Graphical summary. Working model illustrating extracellular nucleosomes promoted microglial ROS production, primed NF-κB signaling pathway, and activated NLRP3 inflammasome via Clec2d and TLR9 in mPFC during chronic stress.

Here, we reported that mice with chronic stress were presented as significant microglial oxidative stress and inflammation, indicating by ROS production, primed NF-κB signaling pathway, activated NLRP3 inflammasome, and upregulated Clec2d and TLR9 in mPFC, together with mushroom-type spine loss, as well as histones and nucleosomes in CSF dictation. Stereotactic injection of nucleosomes was contributed to promote microglial inflammation rather than cell-free histones in mPFC. AAV-Clec2d knocking-down, pretreatment with DNase I to remove DNA, and APC to cleave histones were all effective to inhibit nucleosomes-induced microglial oxidative stress and inflammation. Moreover, AAV-Clec2d knocking-down in mice with chronic stress exhibited limited microglial inflammation, reduced mushroom type spine loss, and improved negative emotional behaviors. The graphic summary was presented in **Figure 9**.

During the recent decade, a surge of clinical and preclinical findings has implicated neuroinflammation as the etiology of stress-related emotional disorders, including anxiety and depression (29). Fast-evolving human neuroimaging techniques provide a more fine-grained understanding of neuroinflammatory response to stress and make it closer to the reality (30). Some preclinical studies have suggested an elevated microglial-derived neuroinflammmation in varied neuropsychiatric disorders by using 18-kDa translocator protein positron emission tomography, which is expressed on activated microglia to quantify central neuroinflammation and has been considered as a promising new validating microglial inflammatory biomarker in psychiatric and neurological disorders (31–33). Mapping studies have identified a cluster of subcortical (basal ganglia, ventral striatum, hippocampus, hypothalamus, and amygdala) and cortical (insula, anterior cingulate, orbitofrontal, ventromedial, and dorsolateral prefrontal cortices) structures that appear central to effects of neuroinflammation on psychological function linked to psychopathology (34, 35). It is worth noting that a diverse array of human and animal studies has demonstrated that mPFC is a key node of cortical and subcortical networks that is critical for the generation and regulation of negative emotion, through its interactions with amygdala, bed nucleus of stria terminalis, periaqueductal gray, hippocampus, and dorsal anterior cingulate cortex (36). However, providing further insight into “how” rather than simply “where” a particular neuroinflammatory process works within the brain during chronic stress is urgently needed. Therefore, we primarily focused on “how” the microglia initiated and accelerated neuroinflammation in mPFC in present study. Our morphological and flow cytometric findings indicated the pro-inflammatory and pro-phagocytic activation of microglia indicated by increased soma volume, retracted processes, ROS production, and upregulated CD68 and also MHC-II expression (37). Moreover, excessive pro-inflammatory and pro-phagocytic activation of microglia is contributed to spine pruning and synaptic transmission impairment, and then leads to development and progression of emotional disorders (38, 39). Similarly, a firmly line of our findings also showed that significantly decreased density of spine, especially mushroom type, in mPFC of mice with CUMS and CORT was observed. Interestingly, Clec2d knocking-down significantly rescued the decrease of mushroom type spine in mPFC of mice with chronic stress to a large extent.

Efforts have been made in the past decades to explore how stress alters microglial function (40, 41). Although it remains far from being revealed which signaling cascade initiates stress-triggered microglial changes, a surge of evidence indicates that several factors, including DAMPs signaling after stress exposure that augments the inflammatory responses, may be involved (42, 43). DAMPs are a variety of intracellular endogenous molecules that released to extracellular space during cellular stress and tissue damage and contribute to several of inflammatory diseases (44). Nowadays, it is largely accepted that DAMPs will initiate sterile immune-inflammation responses by inducing the activation of multiple classic pattern recognition receptors (PRRs), including membrane-bound TLRs and CLRs, cytoplasmic NLRs, and multiple intracellular DNA or RNA sensors, such as TLR3/7/9, cyclic GMP-AMP synthase, absent in melanoma 2, retinoic acid inducible gene I–like receptors, and melanoma differentiation-associated protein 5 (MDA5) (9, 45, 46). Moreover, DAMPs also can be recognized by several non-PRR DAMP receptors, including receptor for advanced glycation end products, triggering receptors expressed on myeloid cells, and several G-protein–coupled receptors and ion channels (47–50). Extracellular histones, damaged nucleosomes, and cell-free DNA are well-known nuclear DAMPs that efficiently induce inflammation, cytotoxicity, and tissue damage. More recently, extracellular histone–induced tissue damage and death have become hot issues of medical concern in sepsis, trauma, ischemia/reperfusion (I/R) injury, and autoimmune diseases (51–55). There are three distinct extracellular forms of histone: free histones, DNA-bound histones (nucleosomes), and neutrophil extracellular traps (NET; webs of extracellular DNA decorated with histones, myeloperoxidase, and elastase). Although, remarkably, throughout current literatures, very little distinction among the presence of different forms of nuclear DAMPs in clinical samples is made, differing significantly their mechanism of instigating inflammation and their cellular and molecular mediators should be identified. Free histones are unique cytotoxic DAMPs that elicit cytotoxicity dominantly through TLR-independent manner (14). Previous human observational studies indicated that the normal value of histones was reported to be about 0.06 ng/ml in serum. However, the levels would dramatically increase as high as 3 ng/ml in multiple trauma patients, and it was associated with coagulopathy, endothelial damage and inflammation, organ dysfunction, and death in sepsis (23). Of note, a previous study reported that lesioned neutrophil-derived extracellular histones H4-mediated cytotoxicity disrupted and lysed the membrane of arterial smooth muscle cells, leading to the destabilization of atherosclerotic plaques (25). Besides, another study identified H1 as a major extracellular DAMPs that caused neurotoxicity within 24 h, even at a low concentration of 50 nM, while the other extracellular core histones H2A, H2B, H3, and H4 did not present neurotoxic at a concentration of 200 nM (24). However, the cytotoxicity induced by extracellular histones does not appear when extracellular histones bind with DNA. A study reported that injection of 1 mg purified nucleosomes did not provoke cytotoxicity in mice, although the saturation of the clearance mechanism had been reached, while injection of 1.25 mg of purified histones is lethal within 1 h in mice (22). Moreover, nucleosomes do not provoke cytotoxicity confirming *in vitro* that isolated nucleosomes did not induce cell death in cultured endothelial cells (13). Similarly, we also confirmed that all five recombinant histones (H1, H2A, H2B, H3, and H4) induced neuronal death at 10 μg/ml (approximately 500 nM) indicated by decreased proportion of Calcein-AM^+^ cells and increased of PI^+^ cells, but not for nucleosomes, even at a relatively high concentration of 15 μg/ml. However, the immuno-stimulation role and pro-inflammatory mechanism of extracellular nucleosomes *in vivo* have not been studied in large detail. Studies wherein incubated nucleosomes in human neutrophils and resulted in activation with upregulating of CD11b and promotion of phagocytosis. Interestingly, the study also indicated that the nucleosome-induced neutrophil activation was equally efficient in both wild-type and TLR2/4 knocking-down mice, indicating that a TLR2/4-independent pathway might be participated in nucleosome-induced inflammation (56, 57). Notably, Clec2d has been identified as a novel receptor that directly binds unmodified poly-basic regions on all histones (N-terminal tail of all histones and also C-terminal tail of H1), providing a potential and attractive therapeutic target for extracellular histones-mediated inflammation and tissue damage. Besides, epigenetic regulation of histones modification also alters Clec2d to recognize them. Studies reported that lysine acetylation blocked recognition by Clec2d, and increasing histone acetylation by inhibiting histone deacetylases (HDAC) reduced the ability of injured cells to stimulate Clec2d (16, 58, 59). In the present study, we confirmed that nucleosomes did induce both Clec2d and TLR9 upregulation. Interestingly, the increase of intracellular Clec2d, especially in endosomes, was larger than that on the membrane, indicating an intracellular trafficking. It is worth noting that the stimulation of Clec2d with nucleosomes did not activate Syk, the downstream signaling kinase of activating CLRs, and Clec2d lacks of immunoreceptor tyrosine–based activation motifs or known signaling adaptors used by other activating CLRs, suggesting that Clec2d was not a signaling receptor. Besides, we only found detectable Clec2d-dependent activation of NF-κB instead of the other MAPKs, including p38, ERK, and JNK on both nucleosomes treatment wild-type and Clec2d knocking-down microglia. Moreover, the nucleosomes-induced microglial oxidative stress and inflammation were abolished in Clec2d knocking-down microglia. Considering that TLR9 is the dominant DNA sensor in endosomes, our results indicated that histones might deliver their bounded DNA to TLR9 in late endosomes to degrade by recognizing Clec2d and then priming TLR9–NF-κB pathway and activating ROS-NLRP3 inflammasome-dependent pro-inflammatory cytokine response. These findings were consistent with previous reports that endosomal TLRs are involved in histones-induced pathology.

There are several limitations in the study. First, extracellular traps, especially for NETs, were typically found in server trauma, sepsis, ischemia-reperfusion injury, cerebral stroke, disseminated intravascular coagulation, and multiple organ failure (14). However, it has not been described in mild stress such as CUMS and CORT. This discrepancies might be derived from the intensity of stress. In the current study, we failed to provide more specific direct morphological evidence on microglial extracellular traps formation (MPO^+^DAPI^+^ microglia) in mPFC and on primary microglia incubated with CORT in a physiological stress concentration (100, 200, 500, and 1,000 ng/ml) (60). The reason might be ascribed to CORT did not induce microglial NETosis. Second, the origin of extracellular nucleosomes is dying or a damaged cell; however, the mechanisms by which nucleosomes are released into the extracellular environment have not been studied in large detail. Existing evidence suggests that nucleosome release shares different mechanisms by different types of cell death, for example, apoptosis, necrosis, pyroptosis, necroptosis, and NETosis. For apoptosis, the active and passive release mechanism of nucleosome is dependent on caspase-activated DNase, as well as Factor VII–activating protease and Factor H activation, respectively (44, 61–63). In contrast to apoptosis, necrotic cell death does not involve the activity of intracellular nucleases but requires circulating nucleases (64). Moreover, in addition to the release of nucleosome/chromatin from dying non-myeloid cells, activated neutrophils (also been found in other cell types, for instance, mast cells, basophils, and macrophages) may undergo NETosis whereby their chromatin is excreted into the extracellular environment to form NETs by a PAD4 activation–dependent manner (65, 66). Third, although we showed the extracellular histones and nucleosomes in CSF, further study investigating nucleosomes secretion presenting in the blood sample of patients with depression will strengthen the relevance of the present study. Finally, the potential sex-specific effect is also needed in further study.

Taken together, our study revealed that extracellular nucleosomes promoted microglial ROS production, primed NF-κB signaling pathway, and activated NLRP3 inflammasome *via* Clec2d and TLR9 in mPFC during chronic stress. An elaborate understanding of the biology of microglial Clec2d on sensing extracellular nucleosomes and histones could be a novel threptic target for chronic stress–related emotional disorders.

## Data Availability Statement

The raw data supporting the conclusions of this article will be made available by the authors, without undue reservation.

## Ethics Statement

The animal study was reviewed and approved by Institutional Animal Care and Use Committee at Xiamen University.

## Author Contributions

HW contributed to conceive the study, designed experiments, interpreted and analysis the data, and wrote the manuscript. HB, CL, QZ, AH, MQ, CHL, and YX conceived the study. GC and LH were the guarantor of this work and, as such, had full access to all data in the study and takes responsibility for the integrity of the data and the accuracy of the data analysis, as well as supervised the work and edited the manuscript. All authors have read and approved this manuscript prior to submission.

## Funding

This work is partly supported by National Natural Science Foundation of China (NSFC 81701091, 81870828, 81801101), Natural Science Foundation of Fujian Province (2020J01017), and Scientific Research Foundation for Personnel, Xiang’an Hospital of Xiamen University (PM201809170003). The funders had no role in study design, data collection and analysis, decision to publish, or preparation of the manuscript.

## Conflict of Interest

The authors declare that the research was conducted in the absence of any commercial or financial relationships that could be construed as a potential conflict of interest.

## Publisher’s Note

All claims expressed in this article are solely those of the authors and do not necessarily represent those of their affiliated organizations, or those of the publisher, the editors and the reviewers. Any product that may be evaluated in this article, or claim that may be made by its manufacturer, is not guaranteed or endorsed by the publisher.
