# Supplementary material for: Extracellular Nucleosomes Accelerate Microglial Inflammation via C-Type Lectin Receptor 2D and Toll-Like Receptor 9 in mPFC of Mice With Chronic Stress
Source: Front Immunol. 2022 Jun 29;13:854202. doi: 10.3389/fimmu.2022.854202 (PMC9276970; doi:10.3389/fimmu.2022.854202)
Supplement: Supplementary file 1 [file DataSheet_1.docx]

**SUPPLEMENTARY MATERIALS**

**SUPPLEMENTARY METHODS**

**Sparse labelling** Diluted rAAV9-CMV-Cre (1:100000 with sterilized PBS; BrainVTA Biomart, Wuhan, China) was mixed with rAAV9-Ef1α-DIO-EGFP-WPRE-pA (BrainVTA Biomart) at a ratio of 1:1, and was bilaterally injected into mPFC (AP, +1.70 mm; ML, ± 0.30 mm; DV, −2.80 mm) at a volume of 100 nl for each hemisphere by using a syringe (Hamilton Bonaduz AG) with micropipette (tip diameter ~15 µm) attached to a KDS LEGATO 130 micropipette puller (RWD Life Science Co., Ltd.) at a flow rate of 0.1 µl per min followed by an additional 5 min to allow diffusion of the virus.

**Cell cytotoxicity staining** Primary neuron and microglia were harvested by FACS and plated in a 12-well plate in complete DMEM (high glucose) media supplemented with 10% heat-inactivated FBS (Gibco) at a density of 2 × 10^4^ cells/well, and then incubated at 37℃ with 5% CO_2_ for overnight. Calcein AM/PI staining (Beyotime Biotechnology) was diluted with buffer (1:1000). A volume of 500 μl staining buffer was added to each well and incubated at 37℃ for 30 min, and then cells were washed by PBS and prepared for imaging by using a confocal laser scan microscope (Zeiss LSM 880 Airyscan; Carl Zeiss Microscopy GmbH) or collected for flow cytometry by using Beckman Cytoflex LX (Beckman Coulter, Inc.).

**Clec2d knocking-down and treatment in primary microglia** Primary microglia were harvested by FACS and plated in a 96-well plate in complete DMEM (high glucose) media supplemented with 10% heat-inactivated FBS (Gibco) at a density of 5 × 10^3^ cells/well, and then incubated at 37℃ with 5% CO_2_ for overnight. For AAV-Clec2d knocking-down, primary microglial were incubated with 10 μl 1×10^8^ VG/ml in 90 μl complete DMEM containing 5 μg/ml Polybrene (Sigma-Aldrich) for 72 h, and 10 μg/ml recombinant nucleosomes (EpiCypher) were incubated for another 12 h, thereafter. TLR9 antagonist E6446 dihydrochloride (0.02 μM; TopScience Co. Ltd.) were pre-treated 12 h before 10 μg/ml recombinant nucleosomes incubation for inhibiting TLR9 activation.

**SUPPLEMENTARY FIGURE LEGEND**

**Supplementary Figure 1 Sparse labelling for spine in mPFC of mice with chronic stress.** A: Working mechanism for sparse labelling were presented. B: Representative immunofluorescence images showing the spine in mPFC of mice in CONT, CUMS, and CORT group were presented. Scale bar: 126×. Spine density, spine length, as well as the number of stubby, mushroom, long thin, and filopodia spine were analyzed. (Two-way ANOVA with *Tukey’s post hoc*, ** *P*<0.01, *** *P*<0.001, compared with CONT. Two replica for n = 3 and 120 spines from n = 3 for the length analysis).

**Supplementary Figure 2 Cytotoxicity of extracellular histones and nucleosomes on primary neuron and microglia.** A: Representative immunofluorescence images showing the CaM^+^ live cells and PI^+^ dead cells for primary neuron incubated with gradient concentration of recombinant nucleosomes and histones for 24 h were presented. Scale bar: 10×. B: Representative flow cytometry images showing the percentage of CaM^+^ live cells and PI^+^ dead cells for primary neuron and microglia incubated with gradient concentration of recombinant nucleosomes and histones for 24 h were presented. C: Survival analysis of primary neuron and microglia incubated with gradient concentration of recombinant nucleosomes and histones were presented (*Kaplan-Meier* survival analysis, n = 3 for each concentration at each given time points). D: Concentration and AUC of IL-1β in primary microglia incubated with gradient concentration of recombinant nucleosomes were presented (One-way ANOVA with *Tukey’s post hoc*, * *P*<0.05, *** *P*<0.001, compared with CONT; ### *P*<0.001, compared with Histones 5 μg/ml; $$$ *P*<0.001, compared with Histones 10 μg/ml; & *P*<0.05, &&& *P*<0.001, compared with Histones 15 μg/ml; ￥￥￥ *P*<0.001, compared with Nucleosomes 5 μg/ml;. n = 3 for each concentration at each given time points).

**Supplementary Figure 3 Clec2d did not involve in MAPK pathway activation with extracellular histones and nucleosomes stimulation in mPFC.** A: Representative western blots showing the expression of microglial p-ERK, p-p38, and p-Jnk on 3 d after histones or nucleosomes stereotactic injection in mPFC with Clec2d knocking-down were presented. B: Relative expression of microglial p-ERK, p-p38, and p-Jnk on 3 d after histones or nucleosomes stereotactic injection in mPFC with Clec2d knocking-down were analyzed (Two-way ANOVA with *Tukey’s post hoc*. n = 3).

**Supplementary Figure 4 Clec2d knocking-down and TLR9 inhibition reduced microglial inflammation.** A: Representative immunofluorescence images showing the mCherry^+^ microglia with AAV-Veh or AAV-Clec2d for 72 h were presented. Scale bar: 10×. B: Concentration of IL-1β in primary microglia with Clec2d knocking-down or/combined with E6446 dihydrochloride were analyzed (One-way ANOVA with *Tukey’s post hoc*, *** *P*<0.001, compared with CONT; ### *P*<0.001, compared with AAV-Veh; $ *P*<0.05, compared with AAV-Clec2d; &&& *P*<0.001, compared with E6446DHC; ￥￥￥ *P*<0.001, compared with AAV-Clec2d+E6446DHC. n = 3).

**Supplementary Figure 5 Clec2d knocking-down reduced microglial activation and inflammation in mPFC as well as improved negative emotional behaviors in mice with CORT.** A: Experimental protocol for mice receiving CORT with Clec2d knocking-down in mPFC, and representative western blots showing the molecular expression were presented. B: Representative histogram images showing CD11b^+^CD45^Low^ microglial ROS in mice receiving CORT with Clec2d knocking-down in mPFC were presented. C: The percentage of CD11b^+^CD45^Low^MHC-II^+^ microglia in mice receiving CORT with Clec2d knocking-down in mPFC were analyzed (Two-way ANOVA with *Tukey’s post hoc*, ** *P*<0.01, *** *P*<0.001, compared with ACSF; ### *P*<0.001, compared with AAV-Clec2d. n = 6). D: Concentration of IL-1β in mice receiving CORT with Clec2d knocking-down in mPFC were analyzed (Two-way ANOVA with *Tukey’s post hoc*, *** *P*<0.001, compared with ACSF; ## *P*<0.01, compared with AAV-Clec2d. n = 6). E: Representative heat maps for ACSF, AAV-Clec2d, AAV-Veh, and CORT group in OF test were presented, and center time% and total distance were analyzed (Two-way ANOVA with *Tukey’s post hoc*, *** *P*<0.001, compared with ACSF; ### *P*<0.001, compared with AAV-Clec2d. n = 12). F: Representative heat maps for ACSF, AAV-Clec2d, AAV-Veh, and CORT group in EPM test were presented, and open arm time% and open arm entries% were analyzed (Two-way ANOVA with *Tukey’s post hoc*, ** *P*<0.01, *** *P*<0.001, compared with ACSF; ### *P*<0.001, compared with AAV-Clec2d. n = 12). G: Representative trajectory images for ACSF, AAV-Clec2d, AAV-Veh, and CORT group in NOR test were presented, and recognition index of investigations and time were analyzed (paired *student’s t* test, ** *P*<0.01, *** *P*<0.001, compared with training. Two-way ANOVA with *Tukey’s post hoc*, ### *P*<0.001, compared with ACSF-testing; $$ *P*<0.01, compared with AAV-Clec2d. n = 12). H: Sucrose preference was analyzed (paired *student’s t* test, *** *P*<0.001, compared with water-water. Two-way ANOVA with *Tukey’s post hoc*, ### *P*<0.001, compared with ACSF-water-sucrose; $$$ *P*<0.001, compared with AAV-Clec2d. n = 12). I: Immobility time% in FS test was analyzed (Two-way ANOVA with *Tukey’s post hoc*, *** *P*<0.001, compared with ACSF; ### *P*<0.001, compared with AAV-Clec2d. n = 12). J: Immobility time% in TS test was analyzed (Two-way ANOVA with *Tukey’s post hoc*, *** *P*<0.001, compared with ACSF; ### *P*<0.001, compared with AAV-Clec2d. n = 12).

**Supplementary Figure 6 Sparse labelling for spine in mice receiving chronic stress with Clec2d knocking-down in mPFC.** A: Representative immunofluorescence images showing the spine in mPFC of mice in CONT, CUMS-AAV-Clec2d, CUMS-AAV-Veh, and CUMS group were presented. Scale bar: 126×. B: Representative immunofluorescence images showing the spine in mPFC of mice in CONT, CORT-AAV-Clec2d, CORT-AAV-Veh, and CORT group were presented. Scale bar: 126×. C: Spine density, spine length, as well as the number of stubby, mushroom, long thin, and filopodia spine were analyzed. (Two-way ANOVA with *Tukey’s post hoc*, ** *P*<0.01, *** *P*<0.001, compared with CONT; ## *P*<0.01, ### *P*<0.001, compared with AAV-Clec2d. Two replica for n = 3 and 120 spines from n = 3 for the length analysis).
