# Supplementary figures and images for: Extracellular Nucleosomes Accelerate Microglial Inflammation via C-Type Lectin Receptor 2D and Toll-Like Receptor 9 in mPFC of Mice With Chronic Stress
Source: Front Immunol. 2022 Jun 29;13:854202. doi: 10.3389/fimmu.2022.854202 (PMC9276970; doi:10.3389/fimmu.2022.854202)

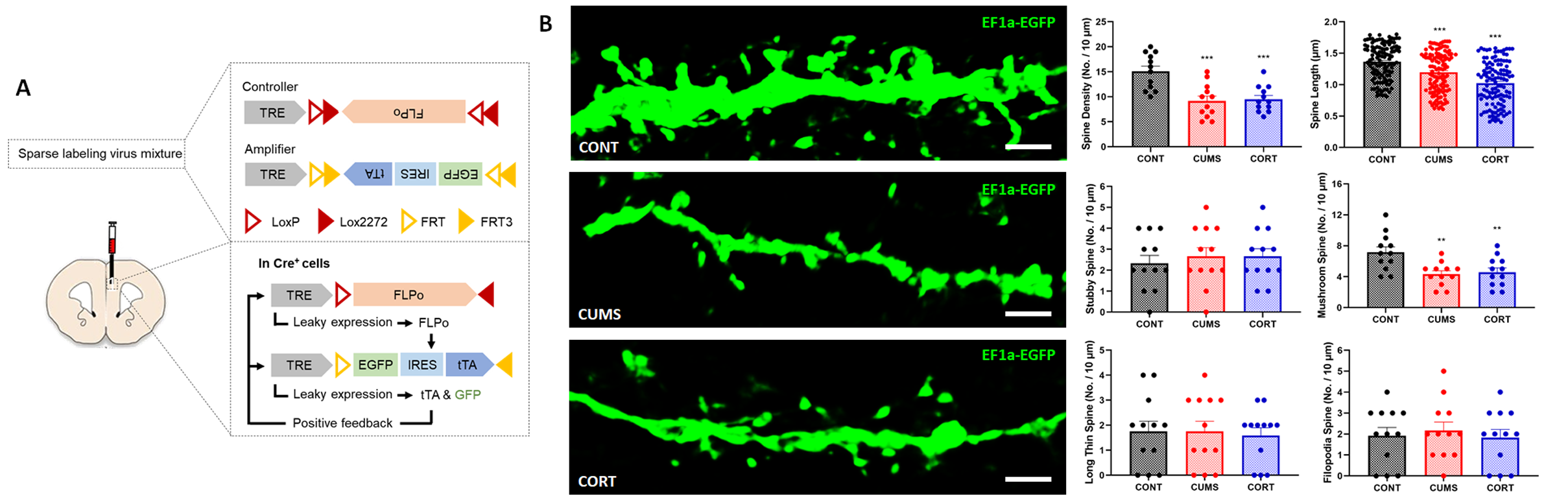

Supplement: Supplementary file 2 [file Image_1.tif]

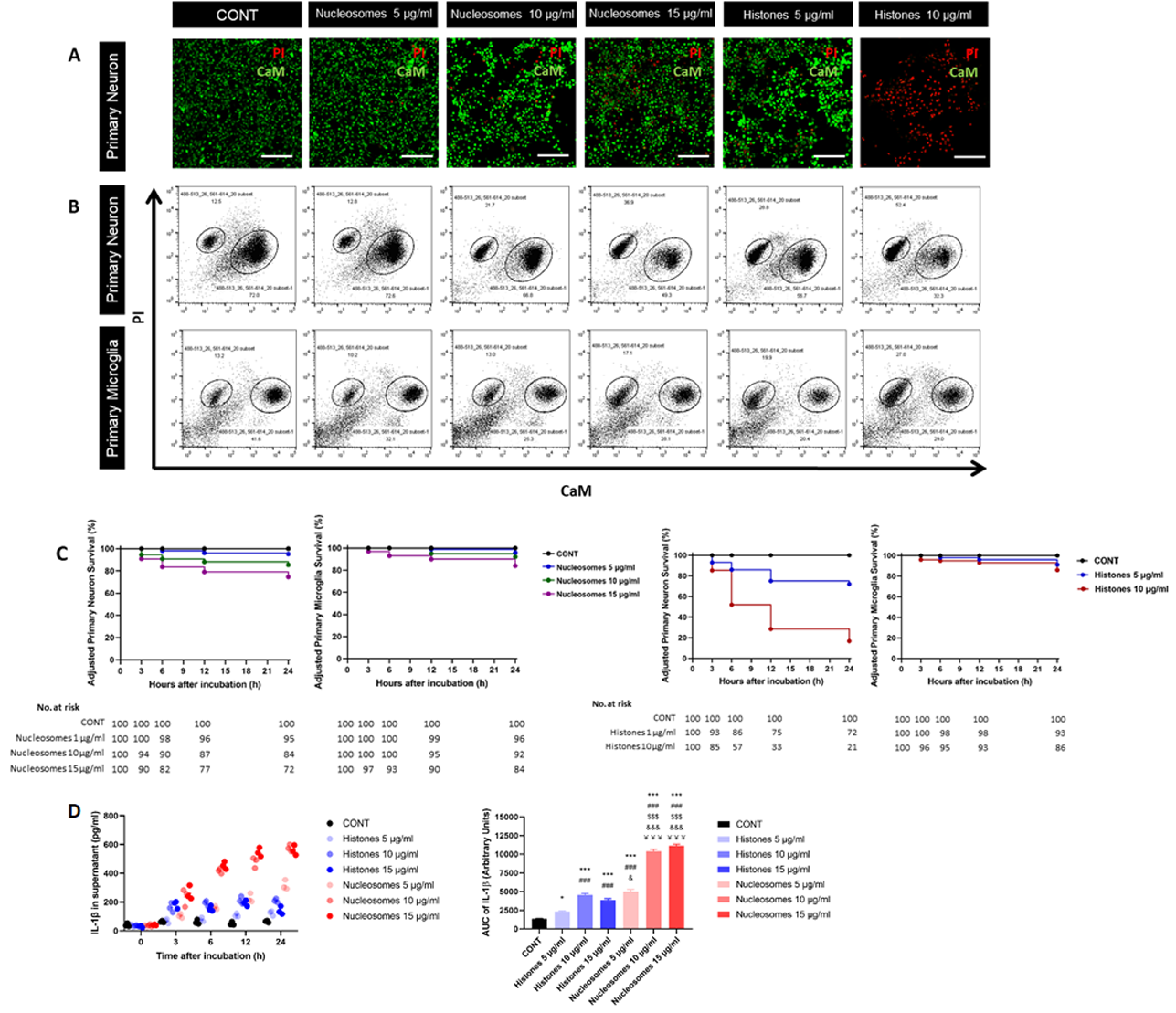

Supplement: Supplementary file 3 [file Image_2.tif]

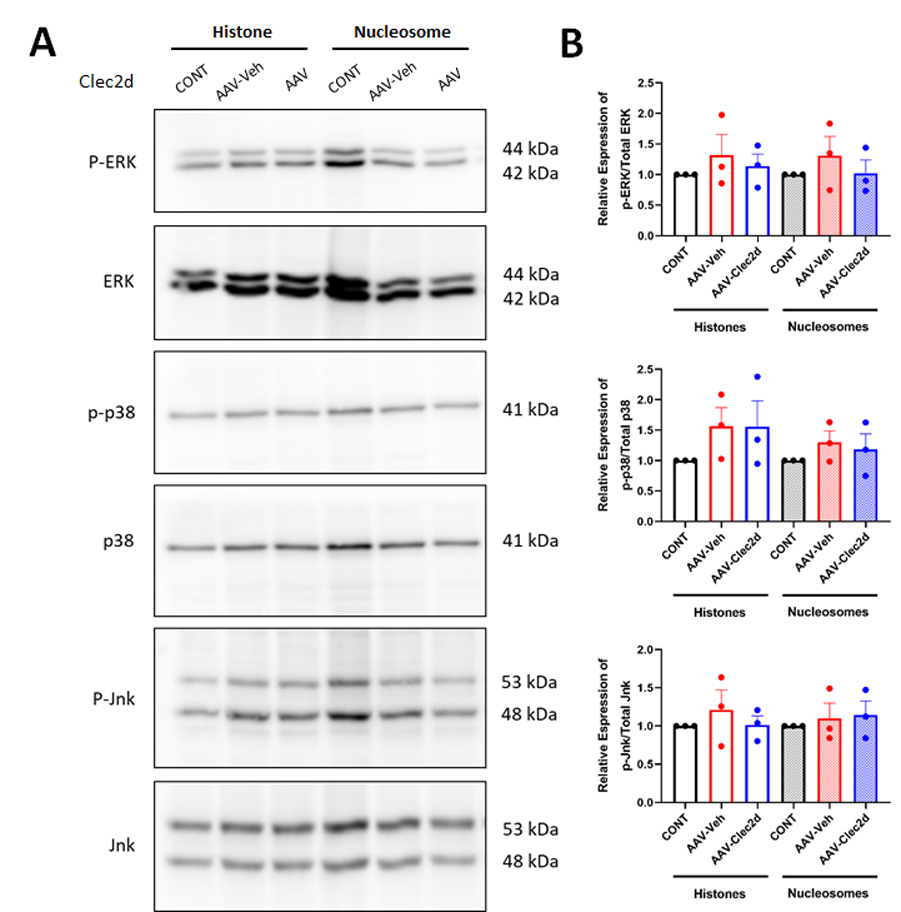

Supplement: Supplementary file 4 [file Image_3.tif]

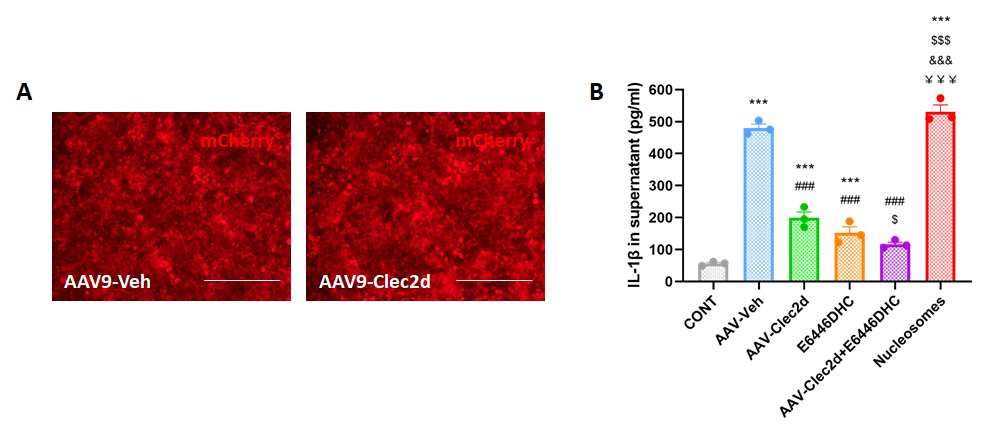

Supplement: Supplementary file 5 [file Image_4.tif]

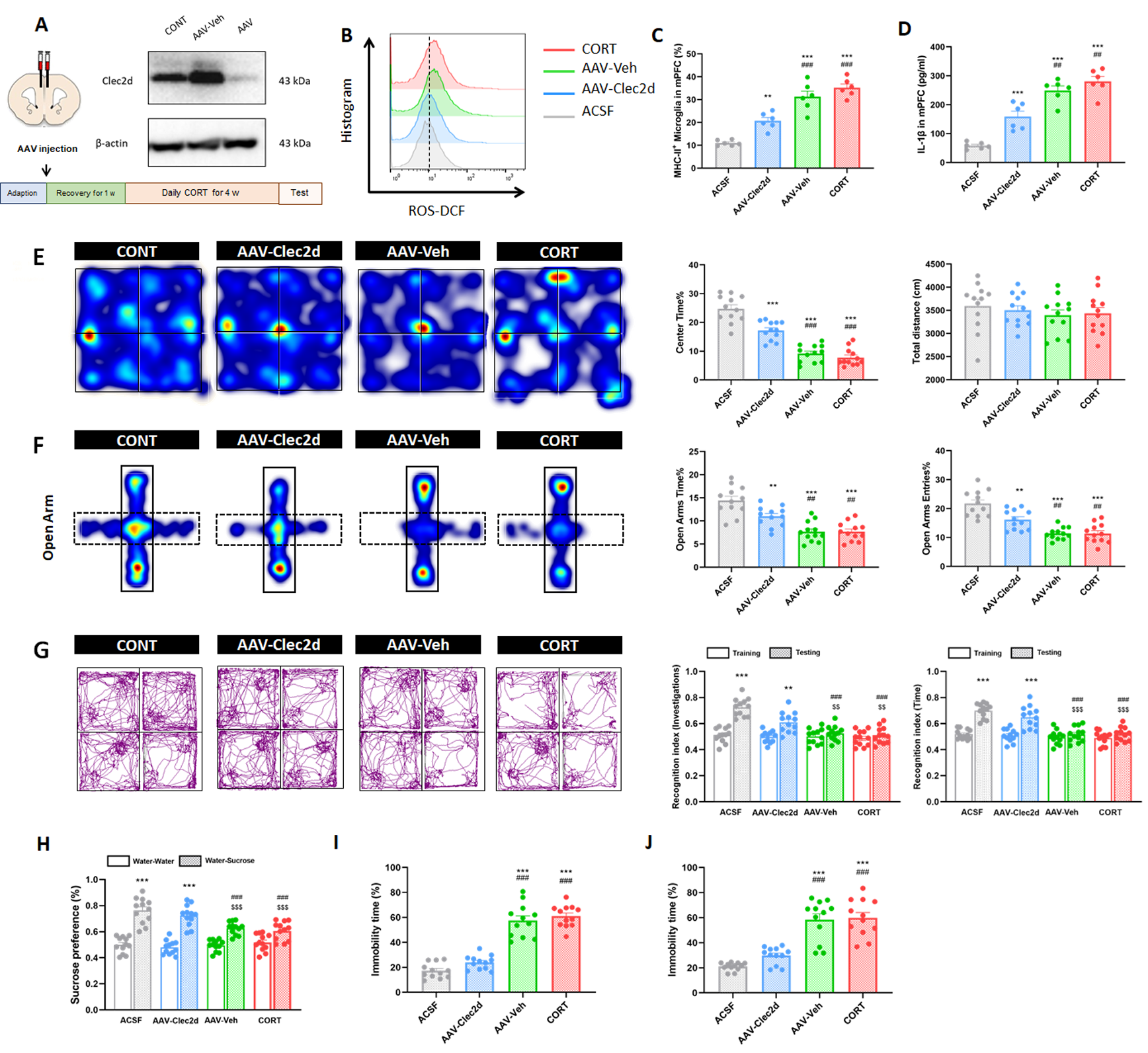

Supplement: Supplementary file 6 [file Image_5.tif]

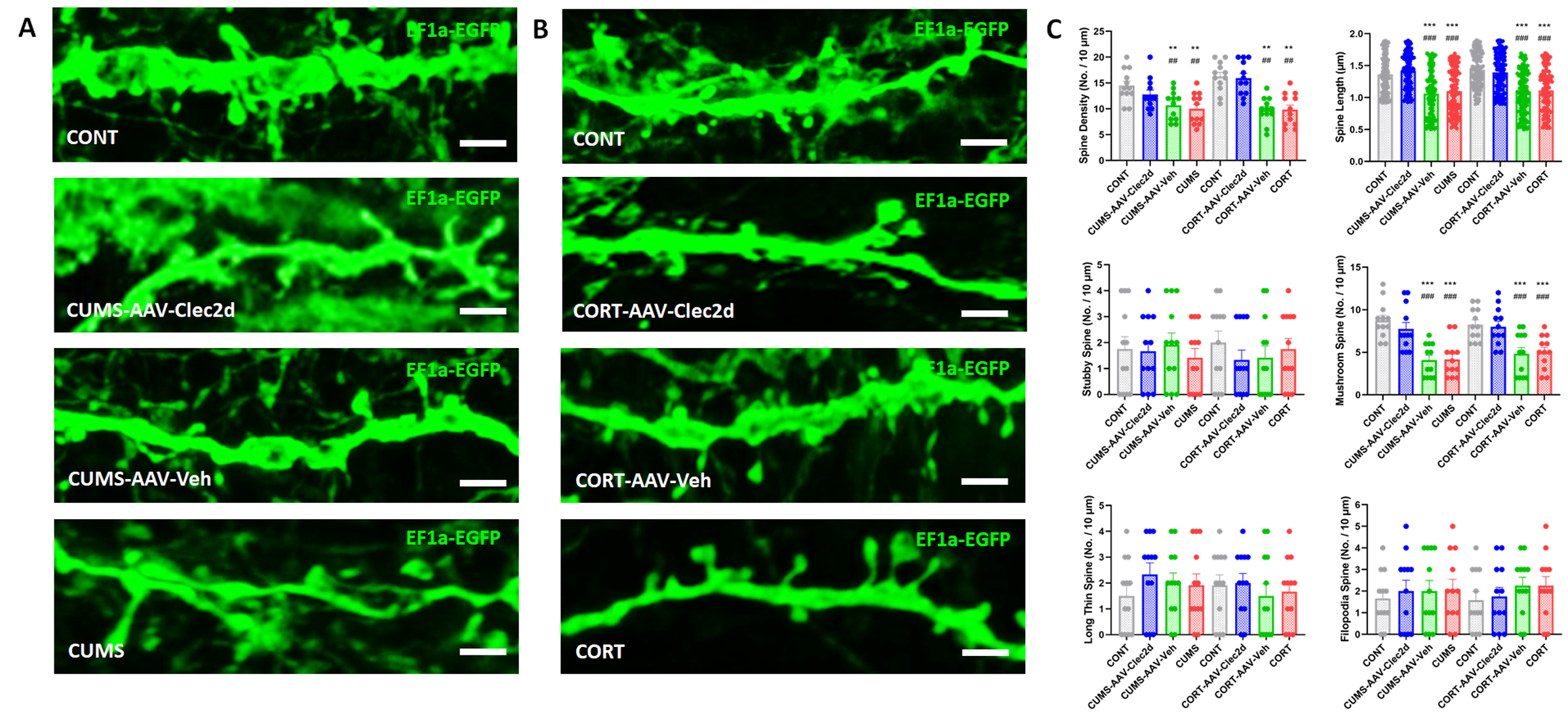

Supplement: Supplementary file 7 [file Image_6.tif]
